# Brazilian guidelines for the pharmacological treatment of pulmonary embolism. Official document of the Brazilian Thoracic Association based on the GRADE methodology

**DOI:** 10.36416/1806-3756/e20240314

**Published:** 2025-05-21

**Authors:** Veronica Moreira Amado, Caio Júlio César dos Santos Fernandes, William Salibe-Filho, Marcelo Basso Gazzana, Ana Thereza Rocha, Hugo Hyung Bok Yoo, Wanderley Marques Bernardo, Suzana Tanni

**Affiliations:** 1. Universidade de Brasília - UnB - Brasília (DF) Brasil.; 2. Grupo de Circulação Pulmonar, Departamento de Cardiopneumologia, Instituto do Coração, Hospital das Clínicas, Faculdade de Medicina, Universidade de São Paulo, São Paulo (SP) Brasil.; 3. Universidade Federal do Rio Grande do Sul - UFRGS - Porto Alegre (RS) Brasil.; 4. Departamento de Saúde da Família, Universidade Federal da Bahia - UFBA - Salvador (BA) Brasil.; 5. Faculdade de Medicina de Botucatu, Universidade Estadual Paulista - UNESP - Botucatu (SP) Brasil.; 6. Programa de Diretrizes, Associação Médica Brasileira, São Paulo (SP) Brasil.; 7. Faculdade de Medicina, Universidade de São Paulo, São Paulo (SP) Brasil.

**Keywords:** Pulmonary embolism, Heparin, Heparin, low-molecular-weight, Factor Xa inhibitors, Fibrinolytic agents, Embolia pulmonar, Heparina, Heparina de baixo peso molecular, Inibidores do fator Xa, Fibrinolíticos

## Abstract

Venous thromboembolism (VTE) is the third most common acute cardiovascular syndrome after acute myocardial infarction and stroke. In recent years, there has been an increase in the incidence of VTE, related to population aging and common comorbidities in the elderly, including chronic cardiorespiratory disease and cancer. On the other hand, disease-related mortality, particularly for pulmonary embolism (PE), shows a decreasing trend, which can be explained by improvements in diagnostic imaging, advances in available therapies, and greater adherence to patient management protocols. The guidelines presented here provide recommendations for the pharmacological treatment of PE in Brazil, on the basis of scientific evidence and with a focus on common practical issues. Six Patient, Intervention, Comparison, and Outcome questions were developed by a group of experts on the topic. Systematic reviews of randomized clinical trials were conducted for each question, with meta-analyses being performed when possible. The level of evidence and strength of recommendation were defined in accordance with the Grading of Recommendations Assessment, Development, and Evaluation approach. With these guidelines, we expect to provide relevant, up-to-date information on the pharmacological treatment of PE.

## RESUMO

O tromboembolismo venoso (TEV) é a terceira síndrome cardiovascular aguda atrás do infarto agudo do miocárdio e do acidente vascular encefálico. Nos últimos anos, observou-se o aumento da incidência de TEV relacionado ao envelhecimento populacional e às comorbidades mais comuns entre os idosos, como doenças cardiorrespiratórias crônicas e neoplasias. Por outro lado, a mortalidade relacionada à doença, em especial ao tromboembolismo pulmonar (TEP), mostra tendência de redução, o que pode ser explicado pelo ganho em capacidade diagnóstica, avanço nas terapias disponíveis e maior adesão a protocolos de manejo desses pacientes. Estas diretrizes têm como objetivo definir recomendações brasileiras para o tratamento do TEP, com base em evidências científicas e com foco em questões práticas e corriqueiras do atendimento médico. Foram elaboradas seis perguntas PICO [pacientes, intervenção, grupo comparador e outcome (desfecho)] por um grupo de especialistas brasileiros no tema. Foram feitas revisões sistemáticas da literatura para cada questão a partir de estudos clínicos randomizados, realizando-se meta-análises quando era possível metodologicamente. O nível de evidência dos trabalhos selecionados e a força das recomendações foram definidos de acordo com o sistema Grading of Recommendations Assessment, Development and Evaluation (GRADE). Com estas diretrizes, espera-se fornecer informações relevantes, atualizadas e com bases científicas sólidas para o tratamento do TEP.

## INTRODUCTION

Venous thromboembolism (VTE) can clinically present as deep vein thrombosis (DVT), pulmonary embolism (PE), or both. In general terms, VTE results from conditions involving hypercoagulability, blood stasis, and endothelial injury (Virchow’s triad). VTE is the third most prevalent acute cardiovascular syndrome, after acute myocardial infarction and stroke. PE has a greater potential for severity and can lead to death either on its own or in association with other comorbidities. In the literature, the incidence of PE ranges from 38 to 99 cases per 100,000 population/year.^1^ In recent years, there has been a trend of increasing incidence of PE; this trend can be attributed to population aging and increased oncological and cardiopulmonary comorbidities in patients > 60 years of age.^2^

Despite the increase in the incidence of PE, the mortality of PE shows a decreasing trend, which is largely explained by improved diagnostic imaging and the emergence of new therapeutic options, such as direct oral anticoagulants (DOACs, including anti-Xa and antithrombin), as well as greater adherence to diagnostic protocols.^2^ ^,^ ^3^

The last recommendation of the *Sociedade Brasileira de Pneumologia a Tisiologia* (SBPT, Brazilian Thoracic Association) for the treatment of PE was published in 2010.^4^ Since then, there have been fundamental changes in therapeutic strategies, an update therefore being required. The objective of these guidelines is to provide updated, practical, and relevant information on the pharmacological treatment of PE and contribute to the advancement of good medical practice in managing PE, which remains a challenge in daily clinical practice. Additionally, it is hoped that these guidelines will assist in discussions with public and private managers regarding treatment options at the hospital and outpatient levels. To this end, the Grading of Recommendations Assessment, Development, and Evaluation (GRADE) approach is the most robust way to address the evidence that is currently available in the literature.^5^

## METHODOLOGY

The current guidelines were developed in accordance with the SBPT. First, a coordinating group was formed, including two specialists in the pharmacological treatment of PE and three experts in evidence-based medicine. Specialists from across the country were invited to join the expert panel. An online meeting was held in April of 2019. After reaching an agreement on the methodology to be employed, the experts were trained in the GRADE approach through written materials and training videos.^6^ After the training, the expert panel formulated questions regarding the pharmacological treatment of patients diagnosed with PE. The questions followed the Patient, Intervention, Comparison, and Outcome (PICO) format. The coordinating group reviewed and adjusted the questions in accordance with the PICO format. The outcomes of interest for each question were defined a priori as critical, important, or not important (Chart 1). 

**Chart 1:** Questions and outcomes included in the present guidelines.

Questions	Critical outcomes	Important outcomes	Minimally important outcome
1. Should home anticoagulation be used in comparison with hospitalization in patients with low-risk PE?	Recurrence of venous thromboembolism; death	Bleeding within 90 days	_
2. Should anticoagulation with DOACs* be used in comparison with anticoagulation with LMWH in PE patients diagnosed with cancer?	Major bleeding**; recurrence of venous thromboembolism; death	Any bleeding	_
3. Should extended anticoagulation be maintained in comparison with placebo or aspirin in patients who have been diagnosed with unprovoked PE and who have completed at least 3 months of anticoagulation?	Major bleeding**; recurrence of venous thromboembolism; death	Any bleeding	_
4. Should treatment with DOACs* be recommended for at least 3-6 months in comparison with conventional anticoagulation (LMWH or UFH, followed by warfarin) in patients diagnosed with low-risk PE, intermediate-risk PE, intermediate high-risk PE after stabilization, or high-risk PE after reperfusion and stabilization?	Major bleeding**; recurrence of venous thromboembolism; death	Any bleeding or clinically significant bleeding	_
5. Should systemic thrombolysis be recommended in comparison with anticoagulation alone (LMWH or UFH) in patients with intermediate-high-risk PE?	Major bleeding**; recurrence of venous thromboembolism; death	Any bleeding; chronic thromboembolic pulmonary hypertension	_
6. Should systemic thrombolysis be recommended in comparison with anticoagulation alone (LMWH or UFH) in patients with high-risk PE?	Major bleeding**; recurrence of venous thromboembolism; death	Any bleeding	_

PE: pulmonary embolism; DOACs: direct oral anticoagulants; LMWH: low-molecular-weight heparin; and UFH: unfractionated heparin. *Apixaban, dabigatran, edoxaban, and rivaroxaban. **Evident bleeding associated with a ≥ 2 g/dL reduction in hemoglobin leading to transfusion of two or more units of blood, occurring in a critical location or organ, or contributing to death.

The team of experts in evidence-based medicine searched for articles and conducted a meta-analysis. The systematic review and meta-analysis was registered with the International Prospective Register of Systematic Reviews (Protocol no. CRD42023481041). The following databases were searched for articles published in English, Portuguese, Spanish, or French and reporting on clinical trials: MEDLINE, EMBASE, and ClinicalTrials.gov. The search terms were defined by the coordinating team, and no date restrictions were applied (supplementary material, Chart S1). 

After selecting the articles, the experts in evidence-based medicine (one of whom is also an expert in managing PE) independently evaluated the titles and abstracts. In case of disagreement, discussions were held until a consensus was reached. Subsequently, the full-text articles underwent qualitative analysis, being evaluated for eligibility. Again, if disagreement occurred, further discussions were held until consensus was reached. The selected articles were then quantitatively evaluated. The reasons for inclusion or exclusion were recorded and presented in accordance with the Preferred Reporting Items for Systematic Reviews and Meta-Analyses (PRISMA) guidelines,^7^ being available in the supplementary material (Figure S1). 

Only results from studies assessing the same intervention were combined in meta-analysis (Chart 2 and Figure S2). For each of the six PICO questions, the quality of the evidence was assessed with GRADE, evidence tables being created with the GRADEpro Guideline Development Tool (McMaster University, Hamilton, ON, Canada).^6^

**Chart 2:** Interpretation of the quality of evidence in accordance with the Grading of Recommendations Assessment, Development, and Evaluation system.^a^

Quality of evidence	Implications	Examples
High (𑁣𑁣𑁣𑁣)	Future research is unlikely to change confidence in the estimated effect; we are confident that we can expect a very similar effect in the population for which the recommendation is intended.	Randomized trials without serious limitations. Well-executed observational studies, with a very large effect.
Moderate (𑁣𑁣𑁣𐊫)	Future research is likely to have an important impact on confidence in the estimated effect and may change this estimate.	Randomized trials with serious limitations. Well-executed observational studies, with a large effect.
Low (𑁣𑁣𐊫𐊫)	Future research is likely to have an important impact on confidence in the estimated effect and will likely change this estimate.	Randomized trials with very serious limitations. Observational studies without special strengths or important limitations.
Very low (𑁣𐊫𐊫𐊫)	Any estimate of an effect is very uncertain.	Randomized trials with very serious limitations and inconsistent results. Observational studies with serious limitations. Nonsystematic clinical observations (e.g., case series and case reports).

a Adapted from the Brazilian National Ministry of Health.^10^

For any given study, the quality of evidence depends on the design, implementation, and risk of bias, and can be classified as high, moderate, low, or very low. The quality may be downgraded by one or two levels when there is evidence of risk of bias, indirect evidence, inconsistency, imprecision, or publication bias. On the other hand, the quality may be upgraded when there is a strong association with the effect without any plausible confounders; when there is evidence of a dose-response relationship; or when all plausible confounders would act to reduce the effect size (Chart 3).^8^ ^-^ ^10^ The methodologists independently assessed the quality of the evidence, and all discrepancies were reviewed until consensus was reached. 

**Chart 3:** Factors affecting the quality of evidence.^a^

Quality of evidence	Situations in which the evidence grade may be reduced	Situations in which the evidence grade may be increased
• High • Moderate • Low • Very low	• Risk of bias • Indirect evidence • Inconsistency • Imprecision • Publication bias	• Strong association, no plausible confounders • Evidence of dose response • Plausible confounders reducing the effect

a Adapted from Guyatt et al.^8^

In February of 2024, the coordinating committee met with members of the expert panel in São Paulo to review all tables containing evidence summaries (Chart S3). Recommendations for each question were made on the basis of critical outcomes, in accordance with the GRADE approach. The recommendations were classified as strong or conditional, depending on the degree of certainty regarding the strength and quality of evidence, among other factors. In accordance with the GRADE approach, we used the term “recommend” for strong recommendations and “suggest” for conditional recommendations. Chart 4 outlines suggested interpretations for all stakeholders, including patients, health care professionals, and policymakers. We used the GRADE evidence to decision framework to organize the discussion and ensure that each of the following factors was considered in formulating the recommendations: balance between desirable and undesirable effects of the intervention; degree of certainty based on the quality of existing evidence; patient values and preferences; resource use implications; and feasibility of implementation (Chart S3).^9^ ^,^ ^10^ Whenever a consensus was not reached, votes were taken, and the results were recorded. 

**Chart 4:** Summary of recommendations for the pharmacological treatment of patients with acute pulmonary embolism.

Questions	Recommendations	Degree of recommendation	Quality of evidence
Should home anticoagulation be used in comparison with hospitalization in patients with low-risk PE?	We suggest home treatment for patients who have been diagnosed with low-risk acute PE and who have guaranteed access to health care services.^a^	Conditional	Very low-quality evidence
Should anticoagulation with DOACs be used in comparison with anticoagulation with LMWH in PE patients diagnosed with cancer?	We suggest treatment with DOACs.	Conditional	Low-quality evidence
Should extended anticoagulation be maintained in comparison with placebo or aspirin in patients who have been diagnosed with unprovoked PE and who have completed at least 3 months of anticoagulation?	We recommend treatment with a DOAC for at least 3-6 months.	Strong	High-quality evidence
Should treatment with DOACs be recommended for at least 3-6 months in comparison with conventional anticoagulation (LMWH or UFH, followed by warfarin) in patients diagnosed with low-risk PE, intermediate-risk PE, intermediate high-risk PE after stabilization, or high-risk PE after reperfusion and stabilization?	We recommend treatment with a DOAC for at least 3-6 months.	Strong	Moderate-quality evidence
Should systemic thrombolysis be recommended in comparison with anticoagulation alone (LMWH or UFH) in patients with intermediate-high-risk PE?	We suggest not using systemic pharmacological thrombolysis.	Conditional	Moderate-quality evidence
Should systemic thrombolysis be recommended in comparison with anticoagulation alone (LMWH or UFH) in patients with high-risk PE?	We suggest systemic pharmacological thrombolysis.	Conditional	Very low-quality evidence

PE: pulmonary embolism; DOACs: direct oral anticoagulants; LMWH: low-molecular-weight heparin; and UFH: unfractionated heparin. ^a^This strategy has as basic premises the following: social support (i.e., understanding of the disease and possible complications; correct use of medications and recognition of potential side effects; and quick access to health care services if needed); and patient preference, a shorter hospital stay being a possibility. Note: Classification of risk for in-hospital mortality or 30-day mortality from acute PE^11^: Patients with a Pulmonary Embolism Severity Index (PESI)^68^ = I or II, or a simplified PESI (sPESI)^69^ = 0. If biomarkers (troponins, B-type natriuretic peptide, or N-terminal pro-B-type natriuretic peptide are negative or normal and there are no signs of right ventricular overload on imaging (CT angiography and/or transthoracic echocardiogram). Patients with a PESI = III-V or an sPESI ≥ 1, as well as signs of right ventricular overload as assessed by elevated biomarkers or imaging. Patients with a PESI = III-V or an sPESI ≥ 1, as well as signs of right ventricular overload as assessed by elevated biomarkers and imaging. Hemodynamically stable patients despite right ventricular overload. Patients with obstructive shock (systemic arterial hypotension-systolic blood pressure [SBP] < 90 mmHg or the need for vasopressors to maintain SBP ≥ 90 mmHg despite adequate volume replacement, and signs of organ dysfunction, including decreased level of consciousness, cold and clammy skin, oliguria/anuria, and/or elevated serum lactate; and/or persistent hypotension-SBP < 90 mmHg or a reduction of 40 mmHg from the usual SBP values for more than 15 min, not related to the onset of arrhythmias, hypovolemia, and/or sepsis, and/or cardiorespiratory arrest).

## Question 1. Should home anticoagulation be used in comparison with hospitalization in patients with low-risk PE?

The possibility of home treatment for low-risk patients with PE has gained traction in recent years with the emergence of DOACs. However, the decision to start home treatment must be based on the following: social support (i.e., understanding of the disease and possible complications; correct use of medications and recognition of potential side effects; and quick access to health care services if needed); and patient preference, a shorter hospital stay being a possibility.^11^

### Evidence

A total of 6,965 articles were identified. Of those, 4,626 were excluded due to the duplicity. Of the remaining 2,360 articles, 2,247 were excluded after title review. The abstracts of 113 articles were read, and 5 articles were selected for inclusion. After discussions between methodologists and specialists, 2 studies were excluded-one because it was a conference abstract and the other because it was not a controlled study. Thus, the meta-analysis included 3 randomized, controlled, open-label studies evaluating early hospital discharge (discharge within the first 24 h of admission in 2 studies and discharge after 24 h of randomization in 1) followed by home treatment for patients diagnosed with acute PE who were classified as having low-risk acute PE in accordance with European Respiratory Society criteria^12^ or the Hestia criteria.^13^ ^,^ ^14^ Two studies initiated treatment with low-molecular-weight heparin (LMWH) and warfarin and assessed noninferiority in comparison with conventional hospital treatment.^12^ ^,^ ^14^ One study initiated treatment with rivaroxaban and compared it with conventional treatment, which was at the discretion of the attending team.^13^

Regarding the critical outcome of recurrent VTE, there was no significant difference between the two strategies (risk difference [RD] = 0.00; 95% CI: −0.01 to 0.01; p = 0.48], with a low level of evidence. Similarly, the critical outcome of death from any cause showed no significant difference (RD = −0.00; 95% CI: −0.01 to 0,01; p = 0.75), with a very low level of evidence (Figure S2.1). 

For the important safety outcome of bleeding of any severity, there was no significant difference between the two treatment strategies (RD = 0.01; 95% CI: −0.01 to 0.02; p = 0.32), with low-quality evidence. The critical outcome of major bleeding was not addressed separately by the selected studies (Figure S2.1). 

### Recommendation

For patients who have been diagnosed with low-risk acute thromboembolism and who have guaranteed access to health care services, we suggest home treatment. This is a conditional recommendation with very low-quality evidence. 

### Comments

Early discharge for home treatment of low-risk PE patients remains a challenge. Although it is an appealing strategy and is recommended in international guidelines,^11^ the concern about recurrence or early deterioration after discharge may pose imminent risks. For the present guidelines, 3 randomized studies were selected. They found no differences in the risk of recurrence or minor bleeding between patients treated at home and hospitalized patients. Among the available therapeutic options, the use of DOACs or LMWH followed by warfarin was assessed. Regarding DOACs, rivaroxaban was compared with conventional hospital treatment in a randomized clinical trial, which resulted in a shorter hospital stay without an increase in adverse events.^13^ Although dabigatran, apixaban, and edoxaban have all been approved for use in patients with acute PE on the basis of studies demonstrating their efficacy and safety in comparison with LMWH and/or warfarin,^15^ ^-^ ^17^ there are currently no randomized clinical trials evaluating these DOACs for home treatment. 

Therefore, for early discharge with home anticoagulation to be a feasible option, certain conditions must be met. These include a low risk of early death or severe complications; no major comorbidities or worsening of concomitant conditions; guaranteed access to the recommended anticoagulant treatment; and guaranteed readmission if symptoms worsen. If these conditions are met, we suggest home treatment for low-risk acute PE. 

## Question 2. Should anticoagulation with DOACs be used in comparison with anticoagulation with LMWH in PE patients diagnosed with cancer?

Venous thromboembolic events are common in cancer patients, and treatment is challenging because of an increased risk of recurrent VTE and because of the risk of bleeding.^18^ Anticoagulation with LMWH for 3 months or longer has been shown to be safer than anticoagulation for the same duration with vitamin K antagonists (VKAs). However, with the emergence of DOACs, the choice of medications for prolonged anticoagulation has been reconsidered. 

### Evidence

A total of 1,381 articles were identified. Of those, 263 were excluded due to the duplicity. Of the remaining 1,118 articles, 1,087 were excluded after title review. The abstracts of 31 articles were read, and 11 articles were fully reviewed. Of those, 5 were excluded. Three were excluded for being duplicate studies, one was excluded for being a post-hoc analysis, and one was excluded for not being in Spanish, French, or English. The meta-analysis included 6 randomized controlled trials evaluating anticoagulation with DOACs in cancer patients with active disease in comparison with standard treatment with LMWH. Two studies evaluated apixaban in comparison with dalteparin.^19^ ^,^ ^20^ One study assessed noninferiority regarding the outcome of recurrent VTE,^20^ and the other assessed superiority regarding any bleeding episode.^19^ Two studies evaluated rivaroxaban in comparison with dalteparin. Both studies had recurrent VTE as the primary outcome, with one using a noninferiority analysis.^21^ ^,^ ^22^ One clinical trial assessed the noninferiority of edoxaban in comparison with dalteparin regarding the composite primary outcome of recurrent VTE or major bleeding.^23^ Finally, one study evaluated the noninferiority of DOACs, for patient convenience, in comparison with LMWH, chosen on the basis of attending physician preference.^24^

Regarding the critical outcome of recurrent VTE, there was a significant difference favoring anticoagulation with DOACs in comparison with LMWH (RD = −0.03; 95% CI: −0.05 to −0.01; p < 0.001), with a moderate level of evidence. The critical outcome of death from any cause showed no significant difference (RD = 0.01; 95% CI: −0.02 to 0.03; p = 0.66), with a moderate level of evidence (Figure S2.2). 

Regarding safety outcomes, the critical outcome of major bleeding showed no significant difference between the therapeutic strategies (RD = 0.01; 95% CI: −0.00 to 0.02; p = 0.21), with a low level of evidence. However, when the important outcome of nonmajor bleeding was evaluated, there was a significant difference favoring conventional treatment with LMWH (RD = 0.03; 95% CI: 0.01-0.05; p = 0.001), with a moderate level of evidence (Figure S2.2). 

### Recommendation

For patients diagnosed with acute thromboembolism and cancer, we suggest treatment with DOACs. This is a conditional recommendation with low-quality evidence. 

### Comments

Cancer patients have a high incidence of venous thromboembolic events,^25^ their risk of developing VTE being 4-7 times as high as that of the noncancer population.^26^ Moreover, 20% of cancer patients develop VTE over the course of their disease,^27^ highlighting a strong relationship between VTE and cancer. Additionally, VTE is the second leading cause of death in cancer patients,^28^ and in up to 10% of cases it may be an indicator of an as-yet unidentified oncological condition.^29^ However, conventional VTE treatment with coumarins has never proven effective for this population. The rate of recurrence and bleeding in cancer patients with VTE is 2-3 times as high as that in the noncancer population.^18^ This is partly due to the difficulty in maintaining cancer patients with VTE within the therapeutic international normalized ratio (INR) range, the results being generally unsatisfactory.^30^ ^,^ ^31^

From the publication of the CLOT study^31^ in 2003 until 2017, LMWHs were the treatment of first choice for cancer-associated VTE.^32^ However, cancer patient adherence to long-term injectable anticoagulant treatment has been shown to be unsatisfactory.^33^ Starting in 2017, studies on DOACs and VTE in cancer patients began to be published. Edoxaban^34^ and rivaroxaban^22^ proved to be superior to dalteparin in preventing VTE but were inferior in terms of bleeding, mainly due to gastrointestinal bleeding in patients with uncontrolled anatomical lesions in the stomach and intestines. Apixaban was shown to be as effective and safe as dalteparin for treating VTE in the cancer population, regardless of the primary site of the lesion.^35^ Although the duration of DOAC use in cancer-associated VTE requires further evidence, most studies have evaluated this condition for a period of 6 months, and this seems to be a reasonable minimum interval for maintaining anticoagulation in this population, provided that the cancer has resolved or has been controlled. 

## Question 3. Should extended anticoagulation be maintained in comparison with placebo or aspirin in patients who have been diagnosed with unprovoked PE and who have completed at least 3 months of anticoagulation?

The decision to maintain anticoagulation for an indefinite period beyond the initial 3 months of treatment after the diagnosis of VTE without a known predisposing factor should carefully consider the associated risks. The recurrence of thromboembolic events represents a significant concern, with an annual incidence ranging from 5.4% to 11% per year after cessation of anticoagulation.^16^ ^,^ ^36^ This risk must be weighed against the potential hemorrhagic complications associated with anticoagulant use. The advent of DOACs has mitigated some of the inconveniences related to the numerous drug and food interactions inherent to VKAs, as well as having eliminated the need for periodic INR monitoring. Moreover, prolonged use of anticoagulants may offer benefits beyond the prevention of VTE recurrence, including a potential reduction in the risk of other cardiovascular complications, such as myocardial infarction and ischemic stroke.^37^

### Evidence

A total of 937 articles were identified. Of those, 48 were excluded due to the duplicity, leaving 889 articles. Subsequently, 867 studies were excluded after title and abstract review. A total of 22 articles were therefore selected for full-text review, with 13 articles being excluded for not meeting the inclusion criteria. Nine articles were discussed among the methodologists, with an additional 4 being excluded: 1 for not providing clear information on the treatment, 1 for not being a controlled study, and 2 for having examined topics that were not relevant to the question at hand. The meta-analysis included 5 randomized controlled trials evaluating extended anticoagulation after at least 3 months of initial anticoagulant treatment. Two studies evaluated warfarin, with a therapeutic INR target between 2 and 3, in comparison with placebo. One study evaluated VTE recurrence as the primary outcome over 24 months of treatment,^38^ and the other study established a combined outcome of VTE recurrence and major bleeding over 18 months of treatment.^39^ One study evaluated three groups: apixaban at the usual dose of 5 mg every 12 h, reduced-dose apixaban at 2.5 mg every 12 h, and placebo for 12 months, with VTE recurrence or death from any cause as the primary efficacy outcome and major bleeding as the primary safety outcome.^16^ Another study evaluated three groups: rivaroxaban at the usual dose of 20 mg daily, reduced-dose rivaroxaban at 10 mg daily, and aspirin at 100 mg daily for 12 months. The primary efficacy outcome in that study was VTE recurrence or death from an unknown cause where PE could not be ruled out as the cause of the fatal event. The primary safety outcome was major bleeding.^40^ Finally, one study evaluated dabigatran at a dose of 150 mg every 12 h vs. placebo for 6 months. The efficacy outcome was VTE recurrence or death from an unknown cause where PE could not be ruled out as the cause of the fatal event. The primary safety outcome was major bleeding or nonmajor but clinically relevant bleeding.^36^

For the critical outcome of VTE recurrence, there was a significant difference favoring prolonged anticoagulation in comparison with placebo (RD = −0.07; 95% CI: −0.10 to −0.04; p < 0.001), with a high level of evidence. The critical outcome of death from any cause showed a significant difference favoring prolonged treatment (RD = −0.01; 95% CI: −0.01 to −0.00; p = 0.02), with a high level of evidence (Figure S2.3). 

Regarding safety outcomes, there were no significant differences between the therapeutic strategies regarding major bleeding, which is considered a critical outcome (RD = 0.00; 95% CI: −0.00 to 0.01; p = 0.14). The level of evidence was high. However, when the important outcome of nonmajor bleeding was evaluated, there was a significant difference between the therapeutic strategies, with a higher number of bleeding events in patients who were anticoagulated for a prolonged period (RD = 0.03; 95% CI: 0.01-0.06; p = 0.01), with a high level of evidence (Figure S2.3). 

### Recommendation

For patients who have been diagnosed with unprovoked PE and who have completed at least 3 months of anticoagulation, extended anticoagulation (for an indefinite period) is recommended. This is a strong recommendation with high-quality evidence. 

### Comments

After a 3-month treatment period for VTE, all patients should be evaluated for extended therapy. Patients who have been diagnosed with VTE and who have a major transient risk factor for thromboembolic events, such as surgeries under general anesthesia lasting longer than 30 min, trauma/fractures, and prolonged hospitalizations with mobility restrictions (longer than 3 days), may discontinue anticoagulation after 3 months of treatment, with a low risk of VTE recurrence.^11^ ^,^ ^41^ For patients with low-risk factors for VTE and a low risk of bleeding, the trend in the literature is to maintain extended anticoagulation, even if the risk factor for thrombosis has been removed; however, this guidance is not consensual.^11^ ^,^ ^41^ For patients who have been diagnosed with unprovoked PE and who have completed at least 3 months of anticoagulation, extended anticoagulation is recommended. For patients with an indication for indefinite anticoagulation, if the bleeding risk is not low, the decision to maintain extended anticoagulation should be individualized and take into account removal of the thrombosis risk factor. 

Extended anticoagulation for an indefinite period is an extrapolation of the positive results from studies evaluating this approach, given that those studies analyzed outcomes within 12-24 months of treatment.^16^ ^,^ ^38^ ^,^ ^39^ Reduced doses of DOACs were assessed in extended treatment studies, showing results similar to conventional doses in terms of effectiveness and safety outcomes, making them an alternative to conventional-dose treatment.^16^ ^,^ ^40^ Periodic reassessment of patients regarding the risk of bleeding during anticoagulation should be conducted using scales specifically designed for this purpose.^11^

If extended anticoagulation is recommended, testing for antiphospholipid antibodies should be included in the diagnostic evaluation of young patients (< 50 years of age), patients with thrombosis in an unusual site, and patients with recurrent thrombosis, as well as those with a history of late pregnancy loss, a history of preeclampsia/eclampsia, or hemolysis, elevated liver enzymes, and low platelet count syndrome.^42^ The diagnosis of antiphospholipid antibody syndrome (APS) implies the use of VKAs instead of DOACs for the prevention of new thrombotic events. This recommendation is based on a study comparing rivaroxaban with warfarin for the prevention of thrombotic events in patients with triple-positive APS (lupus anticoagulant, anticardiolipin, and anti-β_2_-glycoprotein I). The study was prematurely terminated because of an increased risk of thromboembolic events and a higher number of bleeding events in the group treated with rivaroxaban.^42^ ^,^ ^43^

Question 4. Should treatment with DOACs be recommended for at least 3-6 months in comparison with conventional anticoagulation (LMWH or unfractionated heparin [UFH], followed by warfarin) in patients diagnosed with low-risk PE, intermediate-risk PE, intermediate high-risk PE after stabilization, or high-risk PE after reperfusion and stabilization?

For decades, conventional treatment for most PE patients involved initial administration of UFH or LMWH, followed by VKAs. Although this regimen is effective, it is complex because of drug and food interactions associated with VKAs and the need for regular INR monitoring. The development of DOACs such as factor Xa antagonists (rivaroxaban, apixaban, and edoxaban) and thrombin antagonists (dabigatran) has mitigated many of the limitations of conventional therapy, eliminating the need for periodic dose adjustments based on mandatory laboratory monitoring and for dietary restrictions, as well as reducing drug interactions.^44^

### Evidence

A total of 889 articles were identified. Of those, 7 were excluded for being duplicates, leaving 882 articles. Of those, 874 studies were excluded after title and abstract evaluation. Eight articles were selected for full-text review, with 2 articles being excluded for not meeting the inclusion criteria. The meta-analysis included 6 randomized controlled trials evaluating the noninferiority of long-term (3-6 months) treatment with DOACs in comparison with conventional anticoagulation (warfarin with an INR target of 2 to 3). The efficacy outcome was the recurrence of VTE, and safety was assessed by the occurrence of major bleeding or clinically relevant nonmajor bleeding. Two studies assessed dabigatran in comparison with warfarin for a treatment period of 6 months.^15^ ^,^ ^45^ Two studies assessed rivaroxaban vs. warfarin, stratifying patients by treatment periods of 3, 6, or 12 months. The first study evaluated patients with an initial VTE diagnosis,^46^ and the second included patients with symptomatic acute PE.^47^ One study evaluated apixaban vs. warfarin in patients with symptomatic acute VTE for 6 months and followed patients for up to 24 months after randomization.^48^ Finally, one study evaluated edoxaban vs. warfarin for a minimum treatment period of 3 months, with a planned duration of up to 12 months.^17^

Regarding the critical outcome of VTE recurrence, there was no significant difference between DOACs and warfarin (RD = −0.00; 95% CI: −0.01 to 0.00; p = 0.22), with moderate-quality evidence. The critical outcome of death from any cause also showed no significant difference (RD = −0.00; 95% CI: −0.00 to 0.00; p = 0.75), with moderate-quality evidence (Figure S2.4). 

For the critical outcome of major bleeding, there was a significant difference favoring DOACs (RD = −0.01; 95% CI: −0.01 to −0.00; p < 0.001), with moderate-quality evidence. The important outcome of clinically relevant nonmajor bleeding was also favorable to DOACs in comparison with warfarin (RD = −0.03; 95% CI: −0.05 to −0.01; p = 0.003), with moderate-quality evidence (Figure S2.4). 

### Recommendation

For patients with a diagnosis of stable PE or after hemodynamic stabilization, we recommend treatment with a DOAC for at least 3-6 months. This is a strong recommendation with moderate-quality evidence. 

### Comments

On the basis of the results of the studies included in the meta-analysis, which demonstrated noninferior efficacy in comparison with conventional treatment with warfarin and a better safety profile-with fewer major and clinically relevant nonmajor bleeding events-as well as the dosing advantages, even in obese patients^49^ and patients with mild renal dysfunction (creatinine clearance > 30 mL/min),^50^ DOACs have become the outpatient treatment of first choice for PE.^11^ ^,^ ^41^ In Brazil, there are currently no clinical protocol and therapeutic guidelines issued by the Brazilian National Ministry of Health for the management of PE, and the incorporation of DOACs for the treatment of PE in the public health care system occurs heterogeneously, depending on the health departments of the federal states. 

In this context, it is important to highlight conditions in which DOACs are contraindicated, such as in patients diagnosed with APS, for whom warfarin remains the best therapeutic option, and in pregnant or breastfeeding patients, for whom LMWH is the recommended treatment.^11^ ^,^ ^41^ In patients with severe kidney failure (creatinine clearance ≤ 30 mL/min), DOACs should be avoided, the exception being for apixaban, which can be used in such patients. Warfarin is also an alternative for patients with severe kidney failure.^11^ ^,^ ^41^

## Question 5. Should systemic thrombolysis be recommended in comparison with anticoagulation alone (LMWH or UFH) in patients with intermediate-high-risk PE?

Risk stratification of patients with acute PE is crucial for therapeutic planning. Patients without hemodynamic instability (systemic arterial hypotension-systolic blood pressure [SBP] < 90 mmHg or the need for vasopressors to maintain SBP ≥ 90 mmHg despite adequate filling status, and signs of organ dysfunction, including decreased level of consciousness, cold and clammy skin, oliguria/anuria, and elevated serum lactate; and/or persistent hypotension-SBP < 90 mmHg or a reduction of 40 mmHg from the usual SBP values for more than 15 min, not related to the onset of arrhythmias, hypovolemia, and/or sepsis, and/or cardiorespiratory arrest) but who have signs of right ventricular failure on imaging tests, such as echocardiography or chest CT angiography, as well as elevated biomarkers of myocardial injury, such as troponins T and I, and/or right ventricular overload and dilatation, such as B-type natriuretic peptide/N-terminal pro-B-type natriuretic peptide, are considered to be at intermediate-high risk, with an early mortality rate of approximately 9% and a 30-day mortality rate twice as high as that of patients without signs of right ventricular dysfunction.^11^ ^,^ ^51^ ^,^ ^52^ Given the severity of these patients, fibrinolytic treatment could be an alternative with the potential to reduce the risk of hemodynamic instability and death in comparison with conventional treatment with UFH or LMWH. However, the risk of severe hemorrhage, especially hemorrhagic stroke, counterbalances the proposed benefits and must be carefully considered when choosing the treatment.^11^

### Evidence

A total of 1,517 articles were identified. Of those, 22 were excluded because of duplication, leaving 1,495 articles. After evaluation of the titles and abstracts, 1,436 studies were excluded. Fifty-nine articles were selected for full-text review, with 45 articles being excluded for not meeting the inclusion criteria. The remaining 14 articles were jointly evaluated by the methodologists, who excluded 1 article for data analysis without a clear intervention, 1 article for having a different research question, 1 article for being a phase 2 study, and 4 articles for having inclusion criteria that were not relevant to the research question. The meta-analysis included 7 randomized controlled trials comparing peripheral fibrinolysis with conventional anticoagulation for the treatment of patients diagnosed with intermediate**-**high-risk acute PE. 

Three studies compared alteplase combined with UFH or LMWH and anticoagulation with UFH or LMWH alone.^53^ ^,^ ^54^ One of the studies allowed the use of streptokinase as an alternative to alteplase.^55^ In two studies, the primary outcomes were death and hemodynamic deterioration,^53^ ^,^ ^55^ whereas, in one, improvement of right ventricular dysfunction was the primary outcome.^54^ All studies evaluated the occurrence of major or other types of bleeding as safety outcomes. Four studies used tenecteplase as a fibrinolytic drug in comparison with anticoagulation with UFH or LMWH. In three studies, the primary outcome was death or hemodynamic deterioration within 5-7 days of hospitalization.^56^ ^-^ ^58^ One study analyzed mortality at 30 days and 24 months.^59^ The occurrence of bleeding was evaluated by the three studies that analyzed the acute phase. 

Regarding the critical outcome of VTE recurrence, there was no significant difference between fibrinolytic treatment and anticoagulation alone (RD = −0.01; 95% CI: −0.02 to 0.00; p = 0.18), with a moderate level of evidence. The critical outcome of death from any cause did not show a significant difference (RD = −0.00; 95% CI: −0.02 to 0.02; p = 0.12), with a moderate level of evidence. The important outcome of chronic thromboembolic pulmonary hypertension was evaluated in one study only and did not show a significant difference between the two treatment strategies, with a value of p = 0.79. The evidence was considered weak (Figure S2.5). 

There was no significant difference between fibrinolytic treatment and anticoagulation regarding the critical outcome of major bleeding (RD = 0.02; 95% CI: −0.04 to 0.09; p = 0.49), with a moderate level of evidence. The important outcome of nonmajor bleeding tended to occur more frequently with the use of fibrinolytics, but the difference was not significant (RD = 0.13; 95% CI: −0.00 to 0.26; p = 0.06), with a moderate level of evidence (Figure S2.5). 

### Recommendation

For patients with intermediate-high-risk PE, we suggest not using systemic pharmacological thrombolysis. This is a conditional recommendation with moderate-quality evidence. 

### Comments

The management of hemodynamically stable patients with right ventricular dysfunction is challenging in practice because they are relatively common and because approximately 9% of cases have an unfavorable outcome.^52^ The use of diagnostic tools such as bedside point-of-care ultrasound contributes to the characterization and prognosis definition, as well as therapy planning.^60^ Therefore, the use of a more aggressive management strategy that adds risk (especially of bleeding) in comparison with standard treatment with full anticoagulation does not seem to be the best strategy for this particular group of patients. However, fibrinolysis may be beneficial in selected cases, in the context of right ventricular overload and hemodynamic stability. In addition to the combination of right ventricular dysfunction on imaging tests and biomarker alterations (especially when both are abnormal, such as troponins and natriuretic peptides), other parameters may be considered and may contribute to therapeutic decision-making. These include prognostic scores (e.g., the Bova score and the Thromboembolism Lactate Outcome Study score); age; relative hypotension; signs of hypoperfusion (elevated lactate, creatinine, and liver transaminases); concomitant DVT; more severe findings on echocardiography (free thrombus in the right heart chambers) and/or on CT angiography (reflux into hepatic veins and thrombus in the pulmonary arterial trunk or main branches); the need for increased oxygen therapy; severe comorbidities/limited life expectancy; and bleeding risk.^61^

The decision to use a primary, nonsurgical reperfusion strategy in the context of intermediate-high risk (evident right ventricular dysfunction associated with increased afterload caused by PE) should consider the higher likelihood of severe bleeding, particularly in women > 75 years of age.^58^ There are options that can minimize the risk of bleeding, such as using a reduced dose of thrombolytics (via peripheral or catheter-directed administration) and percutaneous pulmonary embolectomy. Some studies suggest that using a reduced dose of thrombolytics (e.g., alteplase at 50 mg over 2 h in comparison with the standard regimen of 100 mg over 2 h via peripheral access) may reduce the incidence of bleeding with similar efficacy.^62^ ^,^ ^63^ A large randomized clinical trial is currently in progress and is expected to shed light on this matter.^64^ There are also data on the use of catheter-directed pulmonary embolectomy in the management of PE, although the current recommendation for this modality is for patients with hemodynamic instability who have contraindications to or did not respond to pharmacological thrombolysis.^63^ Studies have shown hemodynamic improvement with the use of catheter-directed embolectomy, with a low rate of complications.^63^ ^,^ ^65^ The limitations of this technology include the cost of supplies, the availability of the method in hospitals, the expertise of the performing physician, and the logistics required to optimize the time between patient admission and the procedure. However, the absence of control groups treated with anticoagulation (UFH or LMWH) in these studies weakens them methodologically and does not allow a proper assessment of the true benefit of these therapeutic modalities in comparison with conventional treatment. Research with more robust design and outcomes involving these approaches is ongoing. 

## Question 6. Should systemic thrombolysis be recommended in comparison with anticoagulation alone (LMWH or UFH) in patients with high-risk PE?

Patients diagnosed with massive PE can also be classified as being at high risk of death during hospitalization or within 30 days after the acute event. They are characterized by obstructive shock (systemic arterial hypotension-SBP < 90 mmHg or the need for vasopressors to maintain SBP ≥ 90 mmHg despite adequate filling status, and signs of organ dysfunction, including decreased level of consciousness, cold and clammy skin, oliguria/anuria, and elevated serum lactate; and/or persistent hypotension-SBP < 90 mmHg or a reduction of 40 mmHg from the usual SBP values for more than 15 min, not related to the onset of arrhythmias, hypovolemia, and/or sepsis, and/or cardiorespiratory arrest).^11^

Because patients with massive PE are at a high risk of death, they need to be diagnosed quickly in order to initiate reperfusion therapy, with fibrinolysis via peripheral venous access being the most commonly used treatment, in the absence of formal contraindications.^11^

### Evidence

A total of 1,517 articles were identified. Of those, 22 were excluded because of duplication, leaving 1,495 articles. After evaluation of the titles and abstracts, 1,488 studies were excluded. Seven articles were selected for full-text review, with 6 articles being excluded for not meeting the inclusion criteria. Only one article remained for evaluation; therefore, a meta-analysis of the data was not conducted.^66^

The study evaluated only eight patients diagnosed with acute PE, all of whom had hemodynamic instability. The study was terminated early by the research ethics committee, who considered the unfavorable outcome in the control group. Four patients in the intervention group received streptokinase via peripheral intravenous access, whereas the four patients in the control group received UFH. All patients in the control group died during hospitalization. Three patients underwent autopsy, which revealed massive PE and right ventricular infarction, with no significant coronary obstructions in any of them. The patients who received streptokinase were followed for 2 years and showed no signs of pulmonary hypertension or recurrence of PE. No bleeding events occurred in the patients studied. Although fibrinolysis was considered superior to anticoagulation with UFH or LMWH on the basis of the characteristics of the study, the quality of the evidence was very low.^66^

### Recommendation

For patients diagnosed with high-risk PE, we suggest systemic pharmacological thrombolysis. This is a conditional recommendation with very low-quality evidence. 

### Comments

In PE, systemic pharmacological thrombolysis has been recognized as a therapeutic option in cases of hemodynamic instability for many years.^67^ It has been recommended as one of the reperfusion therapy options for high-risk patients.^11^ However, randomized controlled studies supporting this intervention are scarce. 

Because of the criteria used in order to select articles for the present guidelines, only one study was selected. The study by Jerjes-Sanchez et al.^66^ evaluated eight patients: four in the intervention group (who received systemic streptokinase) and four in the control group (who received UFH). All of the patients in the UFH group died. In the intervention group, there were no deaths, and the patients were followed for 2 years without signs of pulmonary hypertension or recurrence of PE. These results, combined with the imminent risk of death, the difficulty in conducting randomized controlled studies with this patient profile, and the availability of fibrinolytic medications in most hospitals, led us to suggest pharmacological thrombolysis for patients with high-risk PE. 

There is, however, a growing number of studies evaluating options for pulmonary reperfusion that carry a lower risk of hemorrhagic complications. These options include catheter-directed thrombolysis, catheter-directed embolectomy, and, in selected cases, surgical embolectomy. These alternatives require infrastructure and a team trained in the procedures, which are costly.^63^
